# Hypertensive Blood Pressure and Its Impact on Functional Outcomes among Older Adults Receiving Comprehensive Geriatric Care

**DOI:** 10.3390/geriatrics8020032

**Published:** 2023-02-28

**Authors:** Marco Meyer, Ulrich Niemöller, Andreas Arnold, Thomas Stein, Damir Erkapic, Patrick Schramm, Christian Tanislav

**Affiliations:** 1 Department of Geriatrics, Diakonie Hospital Jung-Stilling Siegen, 57074 Siegen, Germany; 2 Department of Cardiology, Rhythmology and Angiology, Diakonie Hospital Jung-Stilling, 57074 Siegen, Germany; 3 Department of Neurology, Justus Liebig University, 35385 Giessen, Germany

**Keywords:** comprehensive geriatric care, geriatrics, functional outcome, blood pressure

## Abstract

Background: Comprehensive geriatric care (CGC) is a multiprofessional treatment for older people which considers medical conditions and functional status. The aim of the presented study is to investigate the impact of hypertensive blood pressure (BP) on functional outcomes among older adults receiving CGC. Methods: Functional status was documented by the Barthel index (BI), Tinetti test (TBGT), and timed up and go test (TUG) prior to and after CGC. The results were analyzed in relation to hypertensive BP, indicated by mean BP ≥ 130/80 mmHg determined by 24 h blood pressure monitoring (BPM) while hospitalized. Results: In the presented monocentric, retrospective, observational study, 490 patients were included (mean age (SD): 83.86 ± 6.17 years, 72.2% females). Hypertension in BPM was found in 302 (61.6%) individuals. Hypertensive BP was associated with the female sex (*p* < 0.001) and current fracture (*p* = 0.001), and inversely associated with heart failure (*p* < 0.001), coronary heart disease (*p* < 0.001), atrial fibrillation (*p* < 0.001), urinary tract infection (*p* = 0.022), and hypocalcemia (*p* = 0.014). After CGC, improvements in BI (*p* < 0.001), TBGT (*p* < 0.001), and TUG (*p* < 0.001) were observed in patients with both normotensive and hypertensive BP profiles. The proportion of patients with outcome improvements did not differ between the two groups (BI: 84.4% vs. 88.3%, *p* = 0.285; TBGT: 81.1% vs. 77.7%, *p* = 0.357; TUG: 50.3% vs. 48.4%, *p* = 0.711). Conclusion: Patients both with and without hypertensive BP profiles benefited from comprehensive geriatric care with comparable outcome improvements. Particularly, normotensive BP was associated with chronic cardiovascular comorbidities, indicating increased awareness of the importance of BP management in patients diagnosed with cardiac diseases.

## 1. Introduction

Due to demographic changes, the proportion of older patients with chronic medical conditions and multimorbidity has become increasingly common in developed countries, and is highly associated with health service utilization [1,2]. Comprehensive geriatric care (CGC) as a multiprofessional treatment program was established to address the complex requirements of care of multimorbid older people. It targets medical conditions as well as improving the functional status of the individual patient [3,4,5,6]. As a consequence of geriatric patients’ multimorbidity, several risk factors might affect functional outcomes after CGC. Hypertensive blood pressure (BP) is one of the most common medical diagnoses in older adults, and represents one of the substantial risk factors for disabling cardiovascular events such as myocardial infarction or stroke [7,8]. Hypertension and its decisive cardiovascular complications may potentially lead to a serious functional and cognitive decline, with negative impacts on older patients’ independence and quality of life [8,9]. It is for these reasons that we aimed to investigate the functional outcomes of older adults with hypertensive BP while hospitalized for CGC.

## 2. Methods

### 2.1. Patients

The presented investigation is an observational monocentric retrospective study which was conducted in a large geriatric department (50 beds) in the Diakonie Hospital Jung-Stilling Siegen, in the region of South Westphalia, Germany. The comparison of the baseline data between patients with hypertension in BPM versus those without was conducted using a cross-sectional design. Benefits to functionality after CGC were evaluated using a longitudinal design investigating the cohort of geriatric patients treated in the department.

Patients were selected according to the following inclusion criteria:

(1) Hospitalization for CGC between May 2019 and April 2020;
(2) 24-h blood pressure monitoring (BPM) during their hospital stay;
(3) Complete documentation of outcome parameters (Barthel index, BI; Tinetti balance and gait test, TBGT; timed up and go test, TUG).

All patients who fulfilled the inclusion criteria were selected for further analysis, and the study participants were subdivided into two groups, with and without hypertension, in BPM. Scores from geriatric assessments, including defined outcome parameters (BI, TBGT, and TUG), demographic data, comorbidities, and short-term adverse events during the hospital stay were compared between both groups.

### 2.2. Comprehensive Geriatric Care (CGC)

CGC is a multiprofessional inpatient treatment program specially addressed to older adults. The treatment spectrum in our geriatric department is various; patients with acute medical diagnoses, recovering from surgery, and experiencing typical geriatric syndromes (e.g., immobility, malnutrition, incontinence, chronic pain) are cared for. Patients are assigned to CGC from in-house departments, the emergency room, external hospitals, or their general practitioners. After admission, patients receive an elaborate assessment evaluating mobility, cognitive and emotional capabilities, coping with basic activities of daily living (ADL), and social conditions. BI, TBGT, and TUG were documented upon hospital admission and at discharge as defined outcome scores. Furthermore, Mini Mental Status Examination (MMSE), Shulman’s Clock Drawing test, and the Geriatric Depression Scale (GDS) were carried out upon hospital admission for evaluation of patients’ cognitive and emotional status [10,11,12,13,14]. Hospitalization for CGC was scheduled for 2 weeks, and included the participation of a multiprofessional team of specialized geriatric physicians and nurses; physio-, occupational-, and speech therapists; and psychologists and social workers. Based on detailed evaluation of medical diagnoses and functional deficits, the multiprofessional team developed individualized treatment strategies and discussed treatment progress in a team conference once a week. While hospitalized for CGC, patients received, in total, a minimum of 20 treatment units (30 min each) from the therapeutic team. Every treatment unit consisted of one of the therapeutic measures listed: physiotherapy, logopedics, occupational therapy, or psychological care. The treatment program and therapeutic measures applied were individually adapted to patients’ functional deficits.

### 2.3. Outcome Parameters

#### 2.3.1. Barthel Index

Patients’ ability to cope with basic ADL was expressed by BI that covered 10 items of basic ADL (dressing, walking, grooming, transfer, climbing stairs, using toilet, bathing, bowel/bladder control, and ingestion). The scores were weighted according to the patient’s ability to answer each item independently, with assistance, or dependently. The scores allotted for bathing and grooming were 0 and 5 points; for ingestion, dressing, using toilet, bladder/bowel control, and climbing stairs, 0, 5, and 10 points; and for transfer and walking, 0, 5, 10, and 15 points for each item. In total, BI scores ranged from 0 to 100 points, with high scores suggesting better performance in basic ADL [15,16].

#### 2.3.2. Tinetti Balance and Gait Test

TBGT is a two-component score verifying balance and gait abilities, and fall risk of the older patient. TBGT covers proving balance while sitting and standing; while rising and sitting down on a chair; while a slight nudge given to the patient’s chest; with eyes closed; or while turning 360°, respectively. Gait is assessed by observing gait initiation, the step (length, height, symmetry, continuity), path, and trunk stability. TBGT is rated on a 3-point scale, from 0 to 2 points for each item. The maximum possible TBGT score is 28 points. Higher scores indicate better balance and gait abilities [17].

#### 2.3.3. Timed Up and Go Test

TUG is a widespread assessment tool evaluating walking ability. For TUG, the patient sits in a chair and is asked to stand up, walk three meters, turn around, walk back, and sit down again. The time for TUG completion is measured [18]. In the presented analysis, TUG was subdivided into 5 categories. (5) no walking ability; (4) >30 s for performing TUG test; (3) 20–29 s for performing TUG test; (2) 10–19 s for performing TUG test; and (1) <10 s for performing TUG test.

### 2.4. 24 h Blood Pressure Monitoring

24-h blood pressure monitoring (BPM) is a routinely performed diagnostic procedure in hypertension diagnostics in our geriatric department, and is evaluated by a specialist in internal medicine. If necessary, blood pressure medication is initiated or adapted, respectively, in the clinical care routine. BPM was scheduled for 24 h with wearable devices (custo screen 300, custo med GmbH, Ottobrunn, Germany). Data sets were stored and evaluated in the accompanying archiving system (custo tera, custo med GmbH, Ottobrunn, Germany). Hypertensive BP was defined by BP values ≥ 130 mmHg systolic and/or ≥80 mmHg diastolic, according to the cut-off values for 24 h mean BP in ambulatory blood pressure monitoring, given by the guidelines for the management of arterial hypertension of the European Society of Cardiology and European Society of Hypertension [19].

### 2.5. Data Collection and Statistical Analyses

Demographic parameters; comorbidities; information on short-term adverse events while hospitalized; and the results from the ADL (BI), mobility (TUG, TBGT), cognitive (MMSE and Shulman’s clock drawing test), and emotional assessments (GDS) were documented during the clinical care routine and evaluated for this retrospective, single-center analysis. Normally distributed datasets were presented as mean ± standard deviation (SD), and non-normally distributed data as medians and interquartile ranges (IQR, 25th–75th percentile). Categorical variables were shown as counts and percentages and analyzed via Fisher’s exact test. Normal distribution was verified by the Kolmogorov–Smirnov test. An analysis of the nonparametric data was carried out using the Mann–Whitney U-test for unpaired samples and the sign test for paired samples. In case of normal distribution, a *t*-test was used. Statistical analysis was carried out with PSPP software (version 1.4.1, GNU project).

### 2.6. Ethical Approval

We received ethical approval for this retrospective data analysis (ethical committee of the Medical chamber Westfalen-Lippe and of the Westphalian Wilhelms University, protocol number: 2021-175-f-S).

## 3. Results

**Figure 1:**
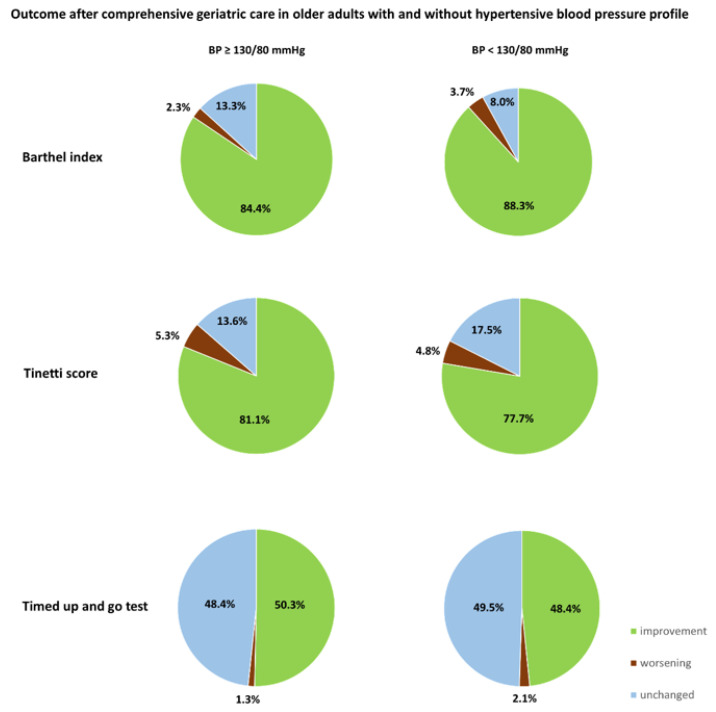
Functional outcomes after comprehensive geriatric care among older adults with and without hypertensive blood pressure (BP) profiles.

In the presented retrospective study, 490 patients were included (mean age ± standard deviation, SD): 83.86 ± 6.17 years). Among them, 354 (72.2%) were female. A hypertensive blood pressure profile (mean BP ≥ 130/80 mmHg) in BPM was found in 302 (61.6%) patients during their hospital stay. The mean BP of all included patients was 135.01 ± 19.07 mmHg systolic and 70.36 ± 10.03 mmHg diastolic. In the subgroup of patients with normotension in BPM (BP < 130/80 mmHg), the mean BP was 115.79 ± 9.07 mmHg systolic and 63.05 ± 6.71 mmHg diastolic. In those patients with hypertension in BPM (BP ≥ 130/80 mmHg), the mean BP was 146.98 ± 12.85 mmHg systolic and 74.91 ± 9.03 diastolic. Diagnoses of heart failure (17.9% vs. 37.2%, *p* < 0.001), coronary heart disease (26.2% vs. 44.7%, *p* < 0.001), atrial fibrillation (23.5% vs. 47.3%, *p* < 0.001), urinary tract infection (9.3% vs. 16.5%, *p* = 0.022), and hypocalcemia (36.1% vs. 47.3%, *p* = 0.014) were more common in patients with normotensive BP, whereas female sex (78.8% vs. 61.7%, *p* < 0.001) and current fracture (55.0% vs. 39.4%, *p* = 0.001) were associated with hypertensive BP profile (Table 1). Upon hospital admission and at discharge, the BI, TUG, and TBGT outcome assessments, as well as the cognitive assessment scores from the MMSE and Shulman’s clock-drawing tests upon admission differed not significantly between both groups. The GDS score was lower in the hypertension group (Table 1). Comparing BI, TBGT, and TUG prior to versus after CGC, patients with and without hypertensive BP benefited from the procedure. In the cohort with normotensive BP, BI increased from the median of 45 (IQR: 31.25–60) to 65 (IQR: 45–80), *p* < 0.001; TBGT from 12 (IQR: 3–18) to 17 (IQR: 11–21), *p* < 0.001; and TUG from a median of 4 (IQR: 3–5) to 3 (IQR: 3–4), *p* < 0.001. In patients with hypertensive BP, BI improved from a median of 50 (IQR: 35–60) to 65 (IQR: 50–80), *p* < 0.001; TBGT from a median of 13 (IQR: 5.75–18) to 17 (IQR: 12–21), *p* < 0.001; and TUG from a median of 4 (IQR: 3–5) to 3 (IQR: 2–4), *p* < 0.001 (Table 2). Overall, the proportion of patients with improvements in BI, TBGT, and TUG did not differ between the groups with and without hypertensive BP while hospitalized (BI: 84.4% vs. 88.3%, *p* = 0.285; TBGT: 81.1% vs. 77.7%, *p* = 0.357; TUG: 50.3% vs. 48.4%, *p* = 0.711) (Figure 1).

## 4. Discussion

In the presented retrospective study, hypertensive blood pressure was found in 61.6% of 24 h blood pressure monitoring examinations in older adults receiving comprehensive geriatric care. Irrespective of hypertensive BP, while hospitalized, patients both with and without hypertensive BP showed improvements in their functional status.

Hypertension is an age-dependent clinical condition with increasing prevalence in older people. Epidemiological data confirm a high prevalence of more than 70% in older adults aged ≥65 years, and the female sex is the predominant gender associated with hypertension in that age group [8,20]. Compared to the presented study, the rate of diagnosis of hypertension upon hospital admission was even higher in the investigated cohort, at 84.7%. This higher prevalence of hypertension could be due to the population consisting of very old (mean age 83.9 years), multimorbid patients. In line with previous data, a hypertensive BP profile was also associated with the female sex in our recent examination [20]. In particular, a hypertensive BP profile in BPM during hospital stay was less common in patients with chronic cardiovascular comorbidities such as heart failure, atrial fibrillation, and coronary heart disease. This association could indicate increased awareness of closely BP monitoring in patients with pre-existing chronic cardiovascular diseases. BP lowering, as a substantial measure for the prevention of serious cardiovascular complications and mortality, has been well-described in previous examinations [21]. A further aspect considered as a cause of lower BP in patients with the aforementioned cardiovascular diagnoses might be heart failure, with disease-related BP decrease as a symptom of low cardiac function and heart failure medication, respectively [22,23]. Both coronary heart disease and atrial fibrillation are risk factors for heart failure development and its consecutive clinical presentation [24,25].

Unlike the lower prevalence of hypertension in patients with cardiac comorbidities, hypertensive BP was associated with a current fracture in the investigated cohort. This point could be explained by several factors that might trigger hypertension. Sympathetic stimulation after surgery with increasing catecholamine release, pain, anxiety, or volume overload could potentially impact the BP profiles of fracture patients [26,27,28,29].

CGC, as a multimodal treatment strategy, also addresses the physical deficits of older people [3,4,5,6]. The results of our investigation revealed that CGC was effective in all included patients irrespective of hypertensive BP profile during their hospital stays, with comparable overall benefits in regard of walking ability, balance, and gait as well as improvement in basic ADL. Thus, hypertension while hospitalized for CGC seems to be of minor importance with regard to functional outcomes, particularly mobility and basic ADL improvements, after the procedure. It could be suspected that serious hypertension-related complications such as suffering a myocardial infarction or a stroke are more relevant to functional decline and potential negative impacts on functional outcomes after CGC as the diagnosis of hypertension itself [9,30,31,32].

Our results revealed lower GDS scores in hypertension patients (median 3 vs. median 4, *p* = 0.017). This statistically objectified difference in GDS between both groups needs to be considered from the perspective that only GDS scores > 5 indicate depression [13].

A strength of this study is the documentation of extensive information on patients’ morbidity and functional status prior to and after the treatment. However, its limitations should also be mentioned. Our investigation was primarily focused on functional outcomes after CGC in the context of hypertension documented in BPM as our standard procedure for assessing patients’ daily BP profiles. Hypertension treatments, or the question of whether a hypertensive BP profile leads to therapy adaptions, were not investigated and could be considered as a major limitation of the presented study. In our specialized geriatric department, BP management is a very dynamic process in the treatment of multimorbid older adults with various diagnoses and comorbidities. Particularly, in patients recovering from surgery, with acute serious injuries or acute medical illnesses BP underlies disease-related fluctuations; thus, several modifications to antihypertensive medications are usually required during the hospital stay. In addition to BPM, daily manual BP measurements were also integrated into the clinical care routine. Initiation or adjustment of antihypertensive medication took place during daily visits, and was rendered by an individual physician’s judgment. To what extent recurrent manual hypertensive BP measurements or hypertension in BPM, respectively, lead to therapy adjustments is not regularly documented. This point affects all patients included in the study. As the second relevant biasing factor, it could be suspected that the white-coat effect impacts patients’ blood pressure profiles due to BPM being performed in a hospital setting. Overall, BPM seems to be advantageous because of the abundant information provided on patients’ daily blood pressure profiles, including night-time readings and short-term BP variability [19].

## 5. Conclusions

Patients both with and without hypertensive BP profiles benefit from comprehensive geriatric care with comparable outcome improvements. Particularly, normotensive BP was associated with chronic cardiovascular comorbidities, indicating increased awareness of the importance of BP management in patients diagnosed with cardiac diseases.

## Figures and Tables

**Table 1:** Factors associated with hypertensive blood pressure profile.

	Total Group (*n* = 490)	Blood Pressure ≥ 130/80 mmHg (*n* = 302)	Blood Pressure < 130/80 mmHg (*n* = 188)	*p*-Value
**Age**	83.86 ± 6.17	83.95 ± 6.47	83.72 ± 5.67	0.684
**Sex**				
Female	354 (72.2%)	238 (78.8%)	116 (61.7%)	<0.001
Male	136 (27.8%)	64 (21.2%)	72 (38.3%)	
**Comorbidities**				
Hypertension	415 (84.7%)	265 (87.7%)	150 (79.8%)	0.020
Heart failure	124 (25.3%)	54 (17.9%)	70 (37.2%)	<0.001
Coronary heart disease	163 (33.3%)	79 (26.2%)	84 (44.7%)	<0.001
Atrial fibrillation	160 (32.7%)	71 (23.5%)	89 (47.3%)	<0.001
Diabetes mellitus	152 (31.0%)	87 (28.8%)	65 (34.6%)	0.192
Chronic obstructive pulmonary disease	46 (9.4%)	24 (7.9%)	22 (11.7%)	0.202
Asthma	9 (1.8%)	5 (1.7%)	4 (2.1%)	0.738
Dementia	100 (20.4%)	62 (20.5%)	38 (20.2%)	>0.999
Depression	57 (11.6%)	36 (11.9%)	21 (11.2%)	0.885
Current fracture	240 (49.0%)	166 (55.0%)	74 (39.4%)	0.001
**Short-term adverse events while hospitalized**				
Delirium	19 (3.9%)	10 (3.3%)	9 (4.8%)	0.473
Pneumonia	18 (3.7%)	8 (2.6%)	10 (5.3%)	0.143
Urinary tract infection	59 (12.0%)	28 (9.3%)	31 (16.5%)	0.022
Hypokalemia	168 (34.3%)	101 (33.4%)	67 (35.6%)	0.626
Hyperkalemia	46 (9.4%)	24 (7.9%)	22 (11.7%)	0.202
Hyponatremia	60 (12.2%)	36 (11.9%)	24 (12.8%)	0.779
Hypernatremia	25 (5.1%)	15 (5.0%)	10 (5.3%)	0.837
Hypocalcemia	198 (40.4%)	109 (36.1%)	89 (47.3%)	0.014
Hypercalcemia	20 (4.1%)	11 (3.6%)	9 (4.8%)	0.640
**Functional assessments**				
Barthel index on admission *	45 (35–60)	50 (35–60)	45 (31.25–60)	0.240
Barthel index at discharge *	65 (50–80)	65 (50–80)	65 (45–80)	0.692
Tinetti on admission *	13 (5–18)	13 (5.75–18)	12 (3–18)	0.573
Tinetti at discharge *	17 (12–21)	17 (12–21)	17 (11–21)	0.502
Geriatric depression scale * (*n* = 431)	4 (2–6)	3 (2–6)	4 (2–6)	0.017
Timed up and go on admission *	4 (3–5)	4 (3–5)	4 (3–5)	0.309
Timed up and go at discharge *	3 (2–4)	3 (2–4)	3 (3–4)	0.279
Mini mental status examination * (*n* = 430)	26 (22–28)	26 (22–29)	26 (22.25–28)	0.901
Shulman’s clock-drawing test * (*n* = 357)	3 (2–4)	3 (2–4)	3 (2–4)	0.706

*: presented as median and interquartile range.

**Table 2:** Barthel index, Tinetti score, and timed up and go test values for geriatric patients with and without hypertensive blood pressure (BP) profiles prior to and after comprehensive geriatric care (CGC).

	Prior to CGC	After CGC	*p*-Value
**Patients with mean BP** **≥** **130/80 mmHg**			
Barthel index (median, IQR)	50 (35–60)	65 (50–80)	<0.001
Tinetti score (median, IQR)	13 (5.75–18)	17 (12–21)	<0.001
Timed up and go test (median, IQR)	4 (3–5)	3 (2–4)	<0.001
**Patients with mean BP < 130/80 mmHg**			
Barthel index (median, IQR)	45 (31.25–60)	65 (45–80)	<0.001
Tinetti score (median, IQR)	12 (3–18)	17 (11–21)	<0.001
Timed up and go test (median, IQR)	4 (3–5)	3 (3–4)	<0.001

## Data Availability

Data supporting the findings of the presented study are available upon reasonable request from the corresponding author.
